# Leaf-cutter ants – mycorrhizal fungi: observations and research questions from an unexpected mutualism

**DOI:** 10.3389/ffunb.2023.1241916

**Published:** 2023-11-16

**Authors:** Michael F. Allen, Hannah Shulman, Philip W. Rundel, Thomas C. Harmon, Emma L. Aronson

**Affiliations:** ^1^ Center for Conservation Biology, Department of Microbiology and Plant Pathology, University of California, Riverside, Riverside, CA, United States; ^2^ Department of Ecology and Evolutionary Biology, University of California, Los Angeles, Los Angeles, CA, United States; ^3^ School of Engineering and Environmental Systems Program, University of California, Merced, Merced, CA, United States

**Keywords:** leaf-cutter ants, mycorrhiza, rainforest, patch dynamics, forest architecture, *Scleroderma sinnamariense*, *Gnetum leyboldii*

## Abstract

Leaf-cutter ants (LCAs) are widely distributed and alter the physical and biotic architecture above and below ground. In neotropical rainforests, they create aboveground and belowground disturbance gaps that facilitate oxygen and carbon dioxide exchange. Within the hyperdiverse neotropical rainforests, arbuscular mycorrhizal (AM) fungi occupy nearly all of the forest floor. Nearly every cubic centimeter of soil contains a network of hyphae of Glomeromycotina, fungi that form arbuscular mycorrhizae. Our broad question is as follows: how can alternative mycorrhizae, which are—especially ectomycorrhizae—essential for the survival of some plant species, become established? Specifically, is there an ant–mycorrhizal fungus interaction that facilitates their establishment in these hyperdiverse ecosystems? In one lowland Costa Rican rainforest, nests of the LCA *Atta cephalotes* cover approximately 1.2% of the land surface that is broadly scattered throughout the forest. On sequencing the DNA from soil organisms, we found the inocula of many AM fungi in their nests, but the nests also contained the inocula of ectomycorrhizal, orchid mycorrhizal, and ericoid mycorrhizal fungi, including *Scleroderma sinnamariense*, a fungus critical to *Gnetum leyboldii*, an obligate ectomycorrhizal plant. When the nests were abandoned, new root growth into the nest offered opportunities for new mycorrhizal associations to develop. Thus, the patches created by LCAs appear to be crucial sites for the establishment and survival of shifting mycorrhizal plant–fungal associations, in turn facilitating the high diversity of these communities. A better understanding of the interactions of organisms, including cross-kingdom and ant–mycorrhizal fungal interactions, would improve our understanding of how these ecosystems might tolerate environmental change.

## 1 Introduction

Interactions between animals and fungi are increasingly recognized as being crucial to ecosystem functioning. Although the role of individual species in structuring communities has long been recognized (Paine, 1966), few examples of belowground cross-phyla animal, plant, and fungal interactions have been described. Leaf-cutter ants (LCAs) in particular are widespread and can be found in Patagonia through the neotropics and into the deserts of Arizona and California and the warm subtropical savannahs of the southeastern United States (Mueller et al., 2017). In addition, they create large belowground structures that alter local carbon flows (e.g., Fernandez-Bou et al., 2019; Swanson et al., 2019; Fernandez-Bou et al., 2020b). Mycorrhizal fungus–plant mutualistic symbioses are even more widespread. They are found almost everywhere globally and play crucial roles in structuring communities and regulating ecosystem processes (Allen, 2022). Is there a direct interaction between mycorrhizal fungi and LCAs affecting community composition and ecosystem functioning? Indirect mycorrhizal fungus–ant interactions have been described, but direct interactions have rarely been documented (Gehring and Bennett, 2009). For example, herbivory by insects can reduce photosynthate allocation to roots and mycorrhizae, thereby reducing mycorrhizal activity and shifting fungal species composition (Gehring and Whitham, 1994). Mycorrhizae can also alter the abundance of both leaf parasites and their predators (Wooley and Paine, 2011), and of aphid populations (Chitty and Gange, 2022), so we would infer that mycorrhizae indirectly interact with ants by affecting their farming of aphids. In meadows and clearcuts within coniferous forests, ants moved mycorrhizal fungal inoculum, thereby facilitating plant recovery following a volcanic eruption (Allen et al., 1984). In semiarid shrublands, harvester ants constructed seed chambers using fine roots containing mycorrhizal fungi (Friese and Allen, 1993), resulting in the rapid formation of arbuscular mycorrhizae (AM) on abandoned nests. By contrast, succession from non-mycotrophic to obligately mycorrhizal plants is slow following other disturbances (Janos, 1980; Allen, 2022). In this study, we pose a novel question: are there LCA– mycorrhizal fungal interactions that have the potential to alter plant community composition?

In lowland neotropical rainforest, the vast majority of studies describing the functioning of mycorrhizae focus on AM, from succession to carbon fluxes (Janse, 1897; Went and Stark, 1968; Janos, 1980; Swanson et al., 2019). We designed instrumentation to study carbon cycling in a neotropical rainforest, which included an automated soil microscopic observation platform coupled to a soil sensor network (Rundel et al., 2009; Swanson et al., 2019). Previously, we demonstrated that LCAs alter gas fluxes between the soil and atmosphere (Fernandez-Bou et al., 2019; Fernandez-Bou et al., 2020a), and, as part of the study of methane production and consumption, we looked at the impacts of LCAs and El Niño phenomena on soil microorganisms (Aronson et al., 2019). From early studies in neotropical forests, an additional issue has always been of interest. We know that ectomycorrhizae (EM), orchid mycorrhizae (ORCM), and ericoid mycorrhizae (ERCM) are widely dispersed across these forests and that EM fungi are common locally in tropical rainforests (Alexander and Lee, 2005). However, we know little about the mechanisms of colonization at the community scale (Helbert et al., 2019; Delevich et al., 2021). Do the impacts of LCAs go beyond localized biogeochemical impacts to affecting the surrounding community? Do LCAs create patches that facilitate the establishment of mycorrhizal communities? Specifically, in this study, using our observatory systems, we asked the following questions: do LCAs import into the nest inocula of mycorrhizal fungi in the form of sporocarpic material, in addition to desirable leaves? In addition, could the physical alterations in nest soils created by the LCAs facilitate new colonization of these communities when nests are abandoned? Our focus was on the LCA nests located within alluvial soils of the La Selva Biological Station. If the mechanisms we propose exist, the importance of interactions between LCAs and mycorrhizal fungi becomes a crucial element in the dynamic stability of these complex communities.

## 2 Background

**Figure 1:**
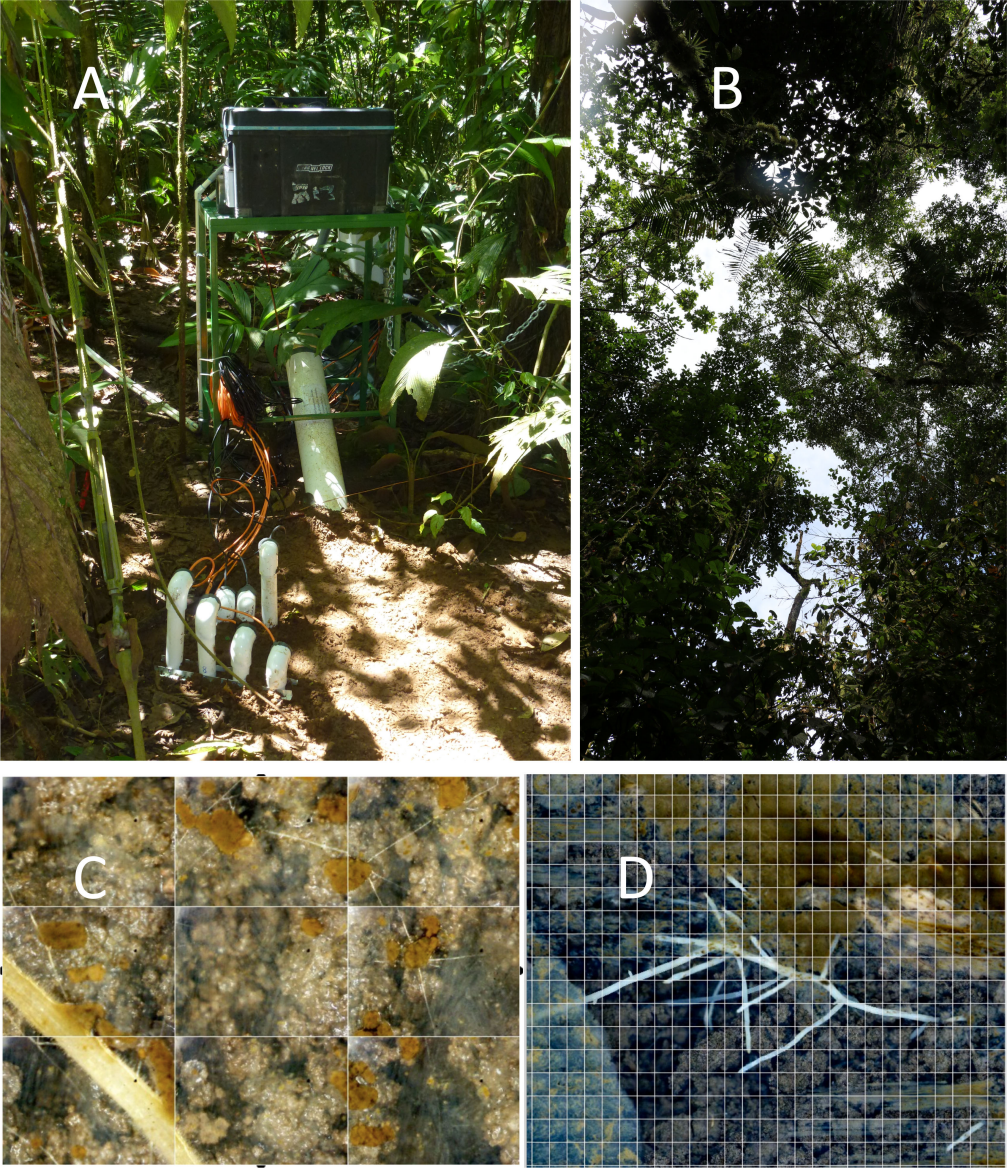
A Soil Ecosystem Observatory (SEO) embedded within a nest footprint with the **(A)** automated minirhizotron in a large white tube and sensors (for CO_2_, O_2_, temperature, and H_2_O) entering the soil showing the sunflecks onto the nest, from **(B)** the canopy gap above the nest. Panel **(C)** is from the control SEO showing an arbuscular mycorrhizal (AM) root with infecting AM fungal hyphae coming from the mycelial network of light-colored fine hyphae, and showing the extensive aggregate development, associated with the AM fungal mycelial network (see text for details). Panel **(D)** shows a root system growing in the abandoned nest. The wall is composed of ant-formed structures. In the abandoned nest, the AM fungal network lags behind the roots, creating openings for new infections.

Lowland neotropical rainforest is composed of a hyperdiverse array of plants, fungi, and animals. The extensive forests in the Central American and Amazon lowlands range from saturated and often standing water conditions, to occasional drier periods tied to El Niño–Southern Oscillation (ENSO) conditions. However, because of the climatic stability, rainfall occurs nearly daily, even during drier periods, and the temperatures often vary as much diurnally as they do seasonally, subject to periodic hot moments of climate events (Fernandez-Bou et al., 2020a). With generally stable environmental conditions, plant diversity can be extraordinarily high. In such situations, mycorrhizal fungal diversity does not necessarily track plant diversity. There exists a lower richness of AM fungi, a monophyletic, ancient group of fungi, the Glomeromycotina, which may predominate because they tend to be less host-specific and functionally more generalist (Allen et al., 1995; Steidinger et al., 2019). Among the vast variety of late-seral plants, AM fungi are well adapted to rainforest conditions (Janos, 1980). Across these hyperdiverse plant communities, communities of AM fungi form extended, interstitial hyphal networks nearly everywhere horizontally, and to several meters deep in the soil (Allen, 2022). In mature rainforests, AM fungi are found in every soil core examined, colonizing both large trees and understory herbs and forming a network occupying nearly every cubic mm (**Figure 1**) of soil observed.

By contrast, there are also low-diversity tropical forest types, such as the high-elevation *Dicymbe* and *Quercus* patches of South America, which are dominated by EM (Nouhra et al., 2019; Delevich et al., 2021), and EM Dipterocarpaceae forests are widespread in Southeast Asia where environmental conditions appear to facilitate a high diversity of EM partners (Helbert et al., 2019). The establishment phase in these plant species-poor patches of EM forests is likely very different from that in high-plant-diversity AM forests. AM fungi show host preferences, but are not known to be species specific (Allen, 2022). EM are often more specific and have repeatedly and independently evolved from many taxa of fungi, ranging from *Endogone* in the Zygomycota to Ascomycota and Basidiomycota. EM plants in the Myrtaceae, Nyctaginaceae, Fagaceae, and some in the Caesalpinioideae form both AM and EM associations. Trees that form dual mycorrhizae, such as *Populus*, *Quercus*, and *Eucalyptus*, will initially form AM on extending roots into an AM network, then transition to EM as individual trees mature (Egerton-Warburton and Allen, 2001; Querejeta et al., 2007; Teste et al., 2020). Other plants form ERCM (primarily Ascomycota), and ORM (Ascomycota and Basidiomycota). Additional detail can be found elsewhere (Allen, 2022). Outside of tropical environments, where the dieback of both plant roots and fungi occurs seasonally, gaps open (Hernandez and Allen, 2013). With drought followed by rainfall events (Taniguchi et al., 2018), or following freeze–thaw events (Allen and Kitajima, 2014), new roots form new mycorrhizae. In seasonal tropical forests, AM predominate, but large disturbances, such as hurricanes, create tree blowdowns and tip-up mounds and thereby open patches that facilitate the establishment of EM (Hasselquist et al., 2011). At smaller scales, animals create disturbances (Aguirre et al., 2021) that facilitate the establishment of new plant–mycorrhizal symbioses. (In this case, EM pines invading into AM shrub/grasslands). However, plants such as the Gnetaceae appear only to form EM, and only with a low richness of fungal partners (Bechem and Alexander, 2011). How do these obligately EM and ERCM plants colonize new patches?

In a lowland neotropical field station, we observed *Gnetum leyboldii* forming widely dispersed patches across the forest. The large seeds are dispersed by water, birds, rodents, monkeys, and even fish (Won and Renner, 2006), and the seedlings form EM. One particular EM fungus observed fruiting in the *G. leyboldii* patch was *Scleroderma sinnamariense*. We do not know how this fungus is dispersed, but no rodent or peccary activity was observed: only that of ants. Wind turbulence in this forest floor is inadequate to disperse spores (Allen et al., 1993; Fernandez-Bou et al., 2020b). From a biodiversity perspective, opportunities to sustain EM and ERCM taxa among patches that shift around the landscape may become important. We know that EM and other mycorrhizae invade and establish themselves in patches within the highly networked AM matrix (Allen, 2022), but we do not how they do so. At extremely small scales, grazing by mesofauna can clip or even graze hyphae from roots, offering some opportunity for colonization (e.g., Fitter and Sanders, 1992), but the existing network is more likely to recolonize than are invading fungi. Disturbance is well known as a critical process in creating patches that allow other species to migrate and establish (Levine and Paine, 1974), thereby increasing community diversity. In tropical rainforests, by opening the canopy, LCA nests create patches of higher temperatures, lower relative humidity (Meyer et al., 2011), and lower nutrient availability (Meyer et al., 2013). These gaps could play a critical role in the ability of plants to reproduce and move by opening the canopy, and facilitating seed germination and photosynthesis (Denslow and Hartshorn, 1994). However, newly established seedlings need both a belowground and aboveground canopy gap. Large, infrequent disturbances (LIDs), such as hurricanes or volcanoes (Dale et al., 1998), can create opportunities for shifting mycorrhizal fungal communities (Hasselquist et al., 2011). However, in addition to these catastrophes, other small-scale disturbances must occur.

Why is this important? Community and ecosystem hierarchies interface where the community composition drives ecosystem processes (Rillig and Allen, 1999). The diverse evolutionary breadth of the fungi forming EM enables them to undertake many biogeochemical processes that AM cannot (Allen, 2022). Some of these EM may be critical for these communities to persist. Small patches of EM plants within an AM matrix are often an important element of these hyperdiverse forests. A key question then emerges: do gaps in the AM fungal network exist? Or does the establishment of EM, ORM, or ERCM, or even the new taxa of AMF, require a large-scale disturbance?

In old-growth neotropical forests, one organism in particular creates soil gaps and canopy gaps. The LCAs in the genus *Atta* create nests that can extend for many meters, opening patches both above and below ground. In the Costa Rican rainforest, nests of the LCA *Atta cephalotes* average 67 m^2^, with 1.8 nests per ha. There are an average of 32 vents per nest, covering a total of 1.2% of the soil surface (Fernandez-Bou et al., 2019). The nests generally have a depth of between 40 cm and 60 cm at the La Selva Biological Station (Swanson et al., 2019). They create gaps that extend up through the canopy, creating chimneys large enough for gas exchange and sunflecks for seedling photosynthesis to occur (**Figure 1**). Internally, AM fungi and root standing crops within the nest is not significantly different from that in the surrounding forest, but the net primary production of roots and mycorrhizal fungi is higher in the nests as turnover is significantly faster (Swanson et al., 2019). The nests turn over approximately on a decadal time scale (Perfecto and Vandermeer, 1993). This very turnover could also provide opportunities for new root–mycorrhizal fungal symbioses if the appropriate inoculum is present.

## 3 Materials and methods

Our study was undertaken at the La Selva Biological Station (Organization of Tropical Studies) in the lowlands of the Caribbean basin of northeastern Costa Rica. The site is in a lowland tropical wet forest, with an average annual rainfall of 4,260 mm (1986–2015) (McDade et al., 1994; Fernandez-Bou et al., 2020a). The average atmospheric temperatures are relatively constant (≈ 25°C). Our site was in an old-growth forest (i.e., older than 1,000 years). The soils at La Selva are derived from volcanic activity, particularly lava flows. At the higher elevations, the volcanic parental material weathered into residual soils. At lower elevations, including in our sites, soils are of alluvial origin, formed in river terraces during the Pleistocene and are classified as Oxisols.

### 3.1 Soil ecological observatories

The nest and control sites were monitored using our Soil Ecosystem Observatory (SEO) (Rundel et al., 2009; Fernandez-Bou et al., 2020a; Rundel et al., in press). An SEO (**Figure 1**) comprises an automated minirhizotron (AMR) composed of a USB-port microscope embedded within a tube (150 cm long and 108 mm in diameter) and mounted on a programmable sled, capable of taking images at 100×. Each image is 3.01 mm wide and 2.26 mm high. Daily scans were taken (up to 44,000 images) and organized into mosaics using RootView software (https://rhizosystems.com/). Surrounding each tube was a network comprising sensors monitoring temperature (T), volumetric water content (θ), CO_2_, and O_2_ at 2-, 8-, 16-, and 50-cm depths at 5-minute intervals, which are summarized here as daily averages (Fernandez-Bou et al., 2020a). The 50-cm depth was below the observable LCA nest construction in these soils. The AMR observations provided insight into the daily activity and to changes in the nest structure, root dynamics, and fungal growth and turnover. Coupling continuous sensor monitoring allowed us to continuously track the dynamics, especially those of temperature and moisture, and couple those drivers with CO_2_ and O_2_ exchange. Together, these measurements represent the drivers (T, θ, and O_2_) and outputs (CO_2_) resulting from the roots and hyphae observed.

Two SEOs were deployed beginning in 2014: one in an LCA nest, and the other in an adjacent undisturbed control site. In 2015, the nest site was abandoned by the LCAs, possibly due to the extreme flooding. However, that SEO remained functional to continue monitoring an abandoned nest. In 2015, two additional active nests were added to the network of SEOs. Sensor data were procured as described in Fernandez-Bou et al. (2020a). Altogether, approximately 40 million images have been observed since 2010. The SEO deployed in the nest is shown in **Figure 1**, including the computer management system, AMR (large white tube), and sensor wires protected within PVC pipes attached to the belowground sensor system (**Figure 1A**). A belowground image from the control AMR (**Figure 1C**) shows the extent of the AM fungal arterial (runner) hyphal network, soil aggregation, and AM roots. **Figure 1D** shows a root growing into the abandoned nest that is not yet colonized by mycorrhizal fungi.

### 3.2 Microbial communities

A campaign in March 2016 was undertaken to describe and measure the microbial communities in control and nest sites. As part of a larger project, we examined sequences from cores taken in nest and control soils to look for the presence of mycorrhizal fungi that might be different in nests compared with those in the surrounding forest matrix. Four 1-m^2^ plots were established, three at SEO sites (one control and two nests), and a fourth additional control site was established in March 2016. Four samples were taken in each of the four quadrats of each plot, for a total of eight control and eight nest samples. A sterilized 2.5-cm corer was used to collect each sample. After collecting, soils were stored at −20°C. To assess the fungal species composition, DNA was extracted from all soils using the MOBIO PowerLyzer™ PowerSoil® kit (Catalog number 12855-100; MOBIO Laboratories Inc., Carlsbad, CA, USA), in accordance with the manufacturer’s instructions.

### 3.3 Sequencing

See Shulmann, Aronson, and Allen, in review, for greater detail and for the assessment of the bacterial and fungal communities. To assess the fungal communities, the internal transcribed spacer 2 (ITS2) region was amplified from soil DNA extracts using the 5.8S-F (5′-AACTTTYRRCAAYGGATCWCT-3′) and ITS4-FunR (5′-AGCCTCCGCTTATTGATATGCTTAART-3′) primer set (Taylor et al., 2016). The DNA was amplified using the Phusion® High-Fidelity Master Mix (NEB), with an additional 3 mM MgCl_2_ and 0.2 μM of each primer. The reaction was carried out using the following thermocycling parameters: initial denaturing at 95°C for 2 minutes, followed by 35 cycles at 95°C for 30 seconds, 55°C for 30 seconds, and 60°C for 4 minutes. The library was sequenced with paired-ended 300-bp reads on the Illumina MiSeq platform. Our original library consisted of 108 samples sequenced on one MiSeq flow cell. This library had been prepared for a related study in which we analyzed the impact of LCA nests on the broader fungal community over time and across different soil types (Shulman et al., in review). Sequence data have been submitted to the National Institutes of Health (NIH) Sequence Read Archive (SRA) under accession number PRJNA749330 (Medicine., 2023).

### 3.4 Bioinformatics

The ITS2 reads were processed using the AMPtk bioinformatics pipeline (Palmer et al., 2018). In brief, USEARCH9 was used to qualify filter and merge paired-end reads, cluster sequences, and pick amplicon sequence variants (ASVs) using the UPARSE algorithm. The ASVs were assigned taxonomy against the UNITE-based AMPtk custom ITS2 database using a hybrid taxonomy assignment approach, which assigns the best match from a combination of global alignment and Bayesian classification. After quality control, there were a total of 3,8639,06 reads and 13,225 ASVs. Our working subset of 16 samples had 720,009 (18.6%) reads and 13,206 ASVs. In our working subset, samples had an average of ≈ 55,000 reads ± ≈ 16,000 reads. In total, 98.3% of taxa were assigned at the phylum level, decreasing to 17.9% assignment and 7.6% assignment at the genus and species levels, respectively.

## 4 Observations and results

### 4.1 Structural observations

The overall architecture of the LCA nests is well described (Swanson et al., 2019; Fernandez-Bou et al., 2020b). They are composed of chambers for the deposition of cut leaves and farming the fungal partners, housing of the queen and offspring, and middens for depositing refuse. Tunnels connect a complex network of chambers and with the outside facilitating CO_2_ respiration (Fernandez-Bou et al., 2020b). Just as importantly, the LCAs remove invading seedlings from the nest footprint to keep open canopies allowing for the penetration of sunflecks (**Figure 1**), thereby increasing T and decreasing θ (**Table 1**). These gaps form chimneys up through the canopy, resulting in drier nest soils during the wet periods of the year and higher rates of CO_2_ efflux (Fernandez-Bou et al., 2019). Below ground, in constructing nests, the LCAs constructed tunnels and chambers where gaps in the roots and hyphal networks opened, providing new areas for plant and fungal colonization in a linked above and belowground patch.

**Table 1:** Conditions of leaf-cutter ant (LCA) nest compared with control locations from the Soil Ecosystem Observatory (SEO).

Depth	T_ave_	T_min_	T_max_	Rainfall^1^		θ (VWC)			T (°C)			^2^CO_2_ (%)			^3^O_2_ (kPa)	
Air	25.4 ± 1.2	22 ± 3.4	31 ± 2.6	12 ± 20	Control	Ab nest	Nest	Control	Ab nest	Nest	Control	Ab nest	Nest	Control	Ab nest	Nest
2 cm					0.50 ± 0.03	0.47 ± 0.03	0.41 ± 0.03	24.6 ± 0.9	26.8 ± 1.4	25.0 ± 0.8	0.51 ± 0.32	0.97 ± 1.09	0.39 ± 0.24	13.0 ± 1.2	14.0 ± 1.21	16.2 ± 0.74
8 cm					0.41 ± 0.03	0.48 ± 0.02	0.41 ± 0.03	24.6 ± 0.8	25.5 ± 0.72	25.1 ± 0.80	2.15 ± 2.2	0.81 ± 0.94	0.48 ± 0.25	ND	ND	15.0 ± 1.2
16 cm					0.43 ± 0.02	0.48 ± 0.02	0.38 ± 0.03	24.6 ± 0.6	25.12 ± 0.66	25.2 ± 0.8	1.42 ± 1.0	1.15 ± 0.89	0.53 ± 0.30	13.8 ± 1.5	15.6 ± 0.58	16.9 ± 1.3
50 cm					0.42 ± 0.01	0.43 ± 0.03	0.30 ± 0.03	24.6 ± 1.2	25.1 ± 0.62	25.8 ± 0.65	1.59 ± 0.91	1.20 ± 0.56	0.99 ± 0.32	15.5 ± 8.0	14.6 ± 1.21	14.1 ± 0.7

^1^The rainfall is the daily total.

^2^%. The atmospheric CO_2_ from Mauna Loa CO_2_ annual mean ranged from 401 ppm (0.0401%) to 408.7 ppm (0.0409%).

^3^kPa. The atmosphere average was 21 kPa.

Ab nest, abandoned nest.

The means ± standard deviations from the control SEO and one of the LCA nests are shown. The weather data (temperature and rainfall) are for the 1,061 days of the study period. The sensor data were daily-averaged for 1,006 days in the control SEO, for 1,043 days in the abandoned nest SEO, and for 695 days in the active nest SEO. Using a paired t-test for each variable (VWC, T, CO_2_, and O_2_), all the variables measured were different among the control, abandoned nest, and active nest locations at a p-value ≈ 0. However, none of the variables were beyond the range of values that would be limiting to the physiology of the roots, fungi, or invertebrates observed (see text for further details).

This architectural change alters the environment experienced by inhabitants of the nest compared with that of the surrounding soil. The nest temperatures (T) are consistently approximately 1°C higher and the soil volumetric water content (θ) is up to 0.12 cm^3^/cm^3^ lower (**Table 1**) than those in the adjacent control SEO. In comparison with the control site, the nests also experience a large decrease in CO_2_ concentration and an increase in O_2_ levels. Those in the abandoned nest were generally intermediate. T, θ, CO_2_, and O_2_ were compared using a paired *t*-test and were all highly significantly different (*p* ≈ 0). However, a few sites were instrumented, and these should be considered pseudoreplicated. On an important biological note, the CO_2_ concentrations measured at all sites were not at toxic levels for the organisms inhabiting them, and O_2_ was not observed to reach anaerobic levels and, therefore, likely did not constrain either invertebrates or fungi.

### 4.2 Organisms in nests

**Figure 2:**
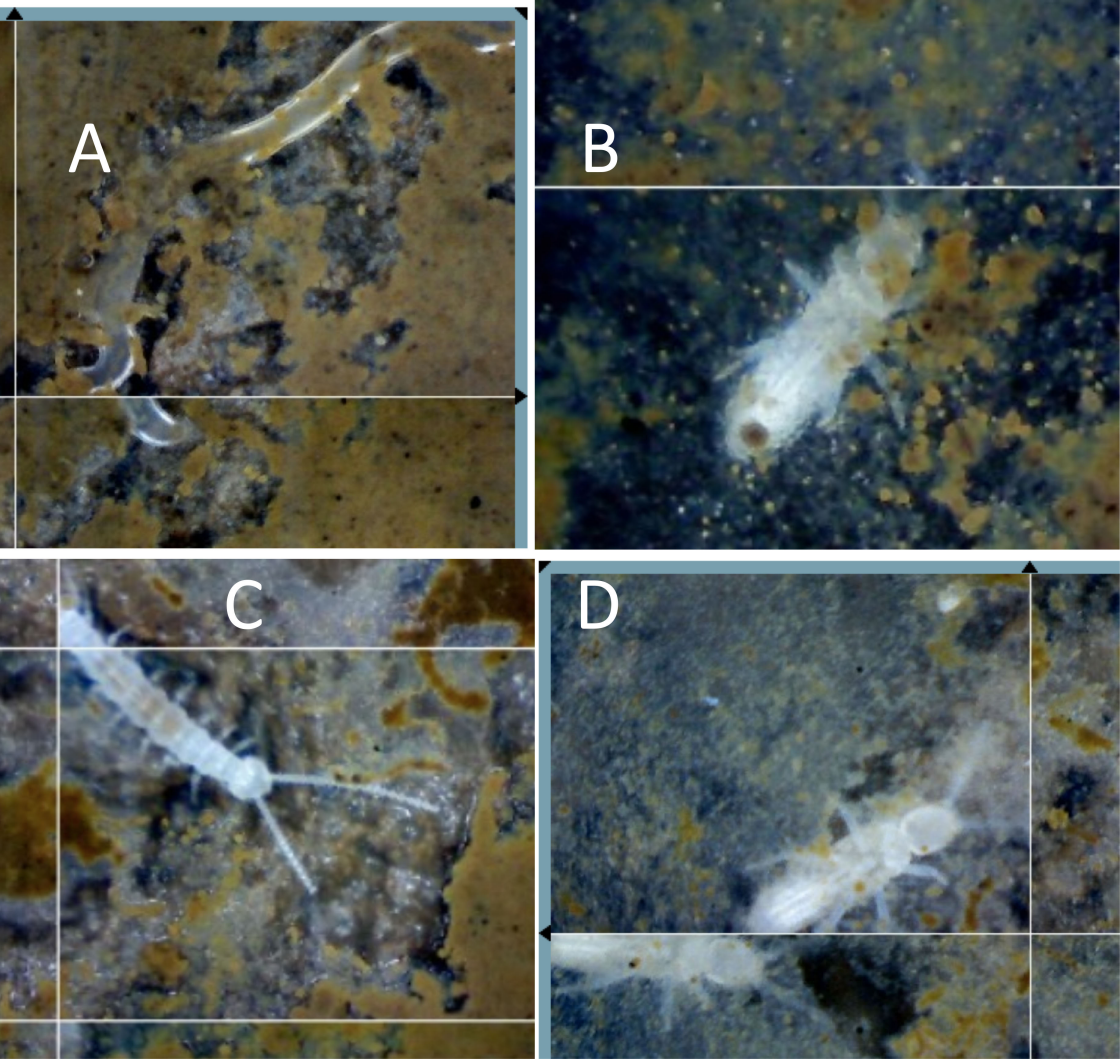
Soil animals within the nest. Decomposers and other invertebrates that are rare in the non-nest soils are commonly found within leaf-cutter ant (LCA) nests feeding on organic materials brought into the nest, or living on the decomposing organic material, including **(A)** a nematode, **(B)** a collembolan, **(C)** a symphylan, and **(D)** insect larvae. Fungal-consuming nematodes have been found with stylets embedded in AM fungal hyphae and spores. Collembola are known to clip AM fungal hyphae and consume EM hyphae, symphylans clip and consume both AM and EM hyphae, and many invertebrates feed on fungi (see text).

Soil animals are extremely rare in the dense clay soils of the matrix outside of nests; we have never observed a nematode, collembolan, or larger soil invertebrate in our SEO in these soils. Within the soil matrix, there may be several reasons for a lack of invertebrates, including the small number of soil pores through which animals can move. The unique environment created by LCA nest architecture opens pores and tunnels for movement and growth of a variety of organisms. Although the standing crop of nest AM fungal hyphal length, 30 mm^–3^, was not significantly different from that of the control AM fungal hyphal length of 32 mm^–3^ (Swanson et al., 2019), the lifespans of individual hyphae were different, with an average hyphal lifespan of 10 days in the nest compared with 32 days around the control SEO. Similarly, fine root length was 3.7 mm^–3^ in the nest, which was similar to the length of 3.5mm^–3^ in the control site, but hyphal lifespan was again far shorter in the nest, averaging 27.5 days in the nest, than the 152 days in the control site. The big difference in the AM root and hyphal lifespans (Swanson et al., 2019) means that there was a greater carbon turnover and more rapid carbon cycling, leading to greater respiration coming from the vent shafts (Fernandez-Bou et al., 2020b). Throughout the active nest tunnels and soil gaps, animals from nematodes to insect larvae were observed (**Figure 2**). This invertebrate activity corresponds to the high rate of turnover of roots and hyphae, as Collembola clip AM fungal hyphae (Fitter and Sanders, 1992; Graf et al., 2019) and symphylans have been observed feeding on both EM and AM fungal hyphae and roots (Allen, 2022).

**Figure 3:**
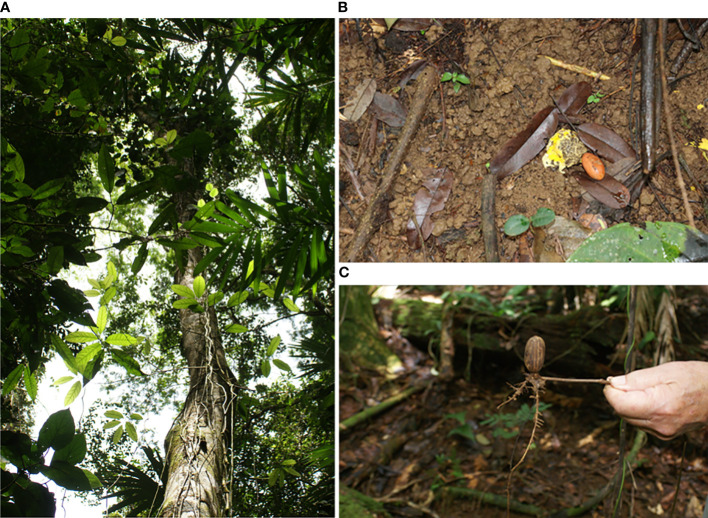
*Scleroderma sinnamariense*, an EM fungus, establishing on a *Gnetum leyboldii* within the footprint of a leaf-cutter ant (LCA) nest. Shown is **(A)** a *G. leyboldii* liana growing up a large tree into the canopy, rooted within an LCA nest matrix with **(B)** a *S. sinnamariense* sporocarp, the peridium of which is being removed and carried away by ants, and **(C)** a germinated *G. leyboldii* seed with ectomycorrhiza (EM) formed with *S. sinnamariense* on the new roots.

The sporocarps of *S. sinnamariense* were found in a nearby patch of *G. leyboldii* (**Figure 3**) that was located within the footprint of an LCA nest. Some components of the periderm and gleba were observed being carried away by ants. *S. sinnamariense* has been described from Costa Rica (Guzmán and Ovrebo, 2000), and this fungus is known to associate with species of *Gnetum* (Tedersoo and Põlme, 2012). The transport of leaves that comprise the substrate for the fungal symbiont of the LCA is readily observable and has been described well elsewhere (Swanson et al., 2019), but other organisms are also abundant in nests that are rare across the soil matrix. Sequencing data (Shulman et al., in review) show that there was a high richness level of fungi in nest and control soils. Additional analyses of the sequence data are still being conducted, but 93% of our ASVs could not be classified at the species level, indicating that marker gene sequencing has drawbacks for the specific identification of ecologically important mycorrhizal species. Our recovered sequences included 226 ASVs of Glomeromycotina, 159 ASVs known to form EM, and at least 135 ASVs that could form ORCM and ERCM. There were distinct differences in the fungal species composition, including those of hypogeous EM fungi. There were also unknown species of the AM fungi Glomeraceae (*Glomus*, *Diversispora*, and *Acaulospora)*, and unknown species of Sebacinaceae, observed to form ERCM (Selosse et al., 2007), and to be present in the roots of many ectomycorrhizal plants (Allen, 2022). Other EM-forming Basidiomycetes (*Tomentella*, *Cenococcum*, and *Phialocephala*, and unknown species of Boletales and Thelephoraceae) were also present. Sequences of *S. sinnamariense* Mont. 1840 (Tedersoo and Põlme, 2012), other hypogeous species of *Scleroderma* spp., and *Rhizopogon* spp., were found within sequences from nest samples but not in control soils. Our search for evidence of mycorrhiza in ITS2 sequencing data suggest that EM, ORCM, and ERCM are rare taxa in tropical fungal communities, yet are present at a surprising level of species richness and may be differentially abundant in nests compared with non-nest-occupied soils.

### 4.3 Abandonment of a nest and reconstruction of the soil matrix

**Figure 4:**
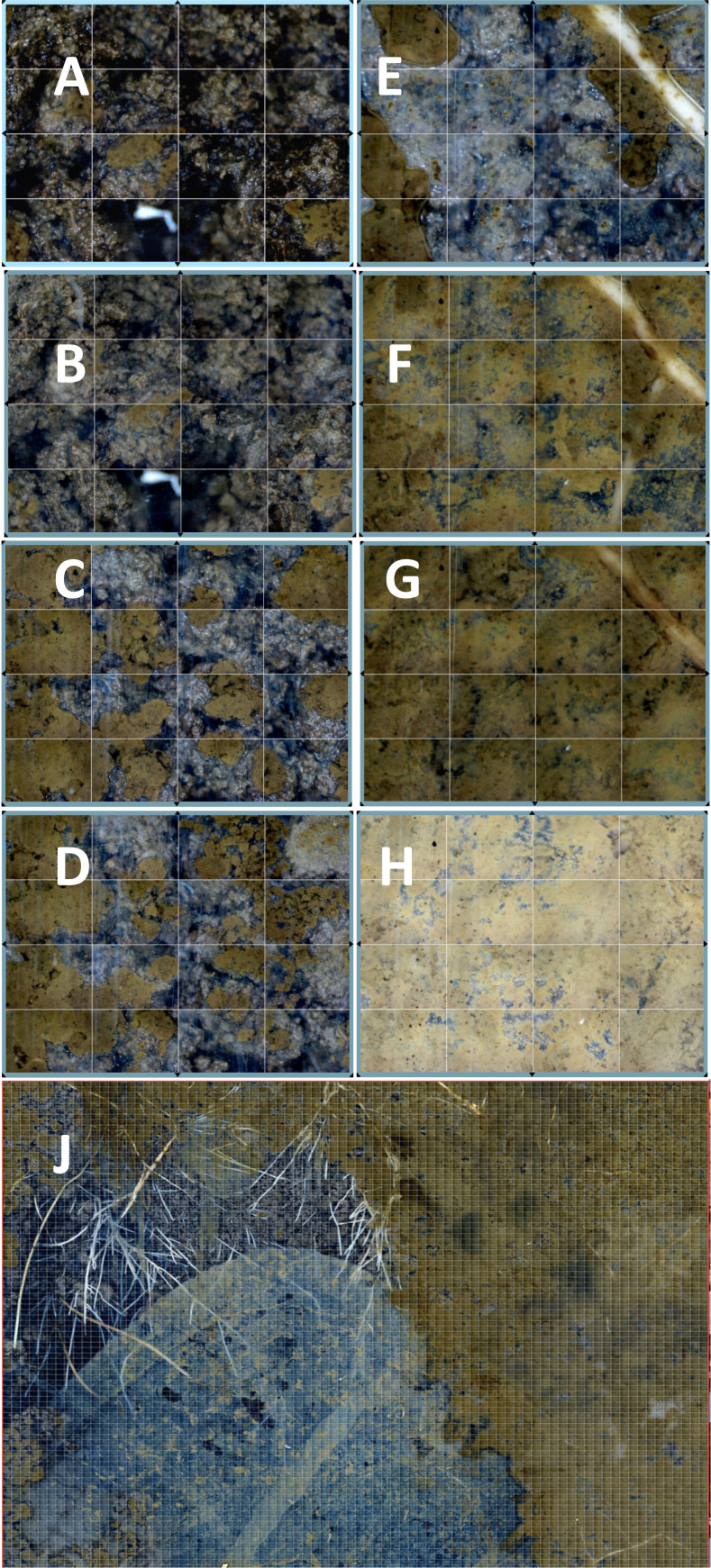
Breakdown of the leaf-cutter ant (LCA) nest following abandonment. The successive breakdown of the wall physical structure during early 2015 after the nest was abandoned, on **(A)** 20 February, **(B)** 21 February, **(C)** 27 February, **(D)** 16 March, **(E)** 20 May, **(F)** 23 June, **(G)** 23 July, and **(H)** 3 December, is shown. The extensive invasion of roots across the matrix **(J)** is shown in the 31 January 2016 mosaic image.

Nests turn over at approximately a decadal time scale (Perfecto and Vandermeer, 1993). Not only is there a noticeable aboveground gap visible during this process, but the chambers and tunnels also collapse (**Figure 4**). The walls, constructed of clay balls and carefully placed by ants, slowly fall apart, turning into wet, clay mush. Roots rapidly grow through the channels and into the chambers. As can be seen in the AMR images, roots are positively geotrophic (**Figure 4J**), whereas mycorrhizal fungi are not (**Figure 4E**). Thus, there is a lag in mycorrhizal formation. However, the conditions within this nest are appropriate for new mycorrhizal formations. One feature of the breakdown is that the tunnels provide initial pathways for gases and invasion pathways for invertebrates and any materials and inoculum that they are carrying. The water and gas composition tends to be intermediate between the nest and control values. From the SEO sensor data, the CO_2_ concentration is intermediate in the abandoned nest compared with the control and nest sites (**Table 1**). For example, at a depth of 16 cm, the mean control CO_2_ concentration for the sampling period was 1.53% (*n* = 1,006), compared with a concentration of 1.18% in the abandoned nest and 0.53% in the active nest. The levels of O_2_ (kPa) were, on average, 13.8% in the control SEO, 15.6% in the abandoned nest, and 16.9% in the active nest (**Table 1**). However, importantly, CO_2_ and O_2_ concentrations do not appear to be limiting to aerobic soil organisms. What does change is the tortuosity, or a reduction in the tortured pathways taken by the gases (Fernandez-Bou et al., 2019); these changes provide passages for animals and for newly ingrowing roots and fungi.

## 5 Discussion

The long-term persistence of communities (animals, plants, fungi, and other microbes) depends in part on an ability to be dynamic in both composition and activity. The tropical rainforest, similar to all other ecosystems, has persisted through variable climates, geological transformations, and biological invasions. The importance of LIDs cannot be underestimated, as hurricanes, volcanoes, earthquakes, and human tillage all transform soils, associated microbes, and plant communities (Lodge and McDowell, 1991). With LIDs, symbionts also invade and change, in part due to soil animal activity (Allen et al., 2018). However, subtle shifts may also occur with small perturbations, facilitating the adjustment of existing communities to less-disruptive change (Morris et al., 2016). For example, global environmental shifts are resulting in elevated CO_2_ and N deposition, along with projected increased temperatures and decreased water availability (Green et al., 2019). For ancient forests to persist, biological symbionts also need to be dynamic in activity and composition. Animals such as LCAs that perturb soils may be especially crucial in maintaining forests and associated microbial species composition.

Similar to human farmers, LCAs create a unique environment that supports the LCA community. They construct “cities” that facilitate the importation of resources (leaves) for an agricultural (in this case, fungal) base. These cities/nests are a network of tunnels that also promote the disposal of wastes, and in the process, facilitate aeration. What other materials might the ants be exploiting? High-protein sources (e.g., fungal sporocarp biomass), unknown vitamins, or trace elements could also be limiting and facilitated by importation. One means of ensuring their self-sufficiency involves LCAs creating disturbances that not only provide for their own survival in a complex ecosystem, but also provide opportunities for increasing the establishment of diverse elements across that community, creating opportunities for shifting other taxa beyond the need for a LID to initiate succession.

Although LCAs select the leaves of particular plant species to move into nests, the criteria for selection remain unknown and other materials may well be utilized (Rockwood, 1976; Masiulionis et al., 2013). We have observed that not all individuals are carrying leaves; ants also carry other materials into the nest. Could some of those other materials be fungal biomass for improving the growth of the *Leucoagaricus* spp. or used as food for the ants themselves? Two materials that have not been addressed are the peridium and the gleba of the sporocarp of fungal fruiting bodies. Masiulionis and colleagues (Masiulionis et al., 2013) observed basidiocarps of *Psilocybe coprophila* being collected by *Acromyrmex lobicornis* LCAs in Argentina, and Allen (2022) observed that peridium and sporocarp top material of *Russula* sp. was cut and transported by ants in a temperate forest. LCA individuals were observed on the *S*. *sinnamariense* sporocarp, and the peridium and gleba of many of the *S*. *sinnamariense* sporocarps were observed being clipped by ants. The gleba and peridium are composed of living hyphae. Fragments of these tissues, along with interspersed basidiospores, could serve as local inoculum. This removal and transport could account for the S. *sinnamariense* DNA material within the nests. Several questions emerge from these observations. Why are LCAs moving sporocarp material into nests? Are they looking for added N due to the high C-to-N ratio of the leaf material? Are there specific nutrients, vitamins, or even antibiotics that outside fungal material provides? Once invertebrates are in the nest, their populations could readily disperse inoculum such as *S. sinnamariense*. Collembola, symphylans, and midge larvae, among the many invertebrates found within the nests, could have readily consumed much of the sporocarpic material while simultaneously redistributing any spores from the sporocarp.

Tropical rainforest communities are predominantly AM (e.g., Allen et al., 1995). The existing AM mycorrhizal fungal networks appear to occupy nearly all of the soil space available, an example of a concept known as community coalescence (Rillig et al., 2016). In EM-monodominant forests, seedlings may acquire the majority of fungi from networks extending from neighboring mature trees (Delevich et al., 2021), but in hyperdiverse AM communities, opportunities for the establishment of EM plants are likely to be limited. In these cases, disturbance gaps become critical to creating opportunities for patches of EM plants, for the invasion of new AM fungi, or even for terrestrial orchid and ericoid mycorrhizal plant and fungal taxa. Therefore, LCA nests may well provide some of these opportunities.

In this study, we have shown that the opportunity exists for LCAs to facilitate transitions in mycorrhizal types and fungi. The ants themselves transport materials, and the chambers and tunnels connect, forming highways for invertebrates, fungi, and roots to navigate the dense clay soil and find nutrients, and enable symbionts to find each other and facilitate gas exchange (Fernandez-Bou et al., 2019; Swanson et al., 2019). The AMR observations suggest that after LCAs abandoned nests, new roots rapidly regrew into any gaps. Roots are positively geotrophic and grow rapidly downward into the reforming soil. In contrast, mycorrhizal fungi are not geotropic, growing in all directions, but are sensitive to the volatile compounds produced by growing root tips (Koske and Gemma, 1992). This means that there is a window of opportunity for any viable fungi brought into contact with newly growing roots, thereby affecting the establishment of a community structure.

The vertical architecture of soil structure and organisms may well be crucial for new mycorrhizal formations. In a sagebrush shrub steppe, roots rapidly grew down before surface inocula could infect them. Newly wind-dispersed inoculum was of limited effectiveness for initiating new mycorrhizae (Friese and Allen, 1991). Therefore, wind-dispersed fungal spores from other locations are unlikely to initiate new mycorrhizal symbioses, as these are deposited on surfaces, where infectable root tips are rarely located. The LCAs and opportunist invertebrates, by bringing EM and other mycorrhizal fungal material deeper into the nest where new roots grow, provide the spatial structure facilitating new mycorrhizal infections. *G. leyboldii*, *S. sinnamariense*, and likely many other EM plant and fungal taxa, may well have narrow associations (Allen et al., 1995; Tedersoo and Põlme, 2012), and two symbionts’ finding each other could lead to the reduced expansion or even survival through stress periods.

The observations of numerous invertebrates within the LCA nests along with the mycorrhizal fungi in comparison with the adjacent non-nest soils suggests that LCA nests are hotspots for biodiversity (Folgarait, 1998; Lavelle et al., 2022). The interactions between ants and mycorrhizal fungi likely go well beyond just LCAs, given the myriad of other taxa burrowing into soil in a similar manner, albeit on a smaller scale. Far more research into both community and ecosystem functioning and biological interacting networks is an opening into a far more complex world than even ecologists have acknowledged.

In scaling up, we need a better spatial understanding of the roles played by EM, ERCM, and ORM in tropical rainforest ecosystems, which are dominated by AM plants, and a better sense of the time scales on which these dynamic ecosystems function. A better understanding of these processes will allow us to better predict and manage these crucial ecosystems as our global environment changes. Using cores allows us to gain insight into ecosystem processes. However, only an infinitesimally small amount of the forest can be sampled from cores at infrequent intervals. For example, in the most comprehensive survey to date of fine-root standing crop (Espeleta and Clark, 2007), conducted across 18 plots that were 0.5 ha in size using a 47.4-mm diameter corer, only approximately 0.001% of the surface area was actually analyzed. Our SEO increased the temporal resolution but at a limited number of locations. Coupling campaigns and SEO observations increased our spatial and temporal resolution, but the number of data to be processed and understood also dramatically increased. Newer approaches are needed to merge sensing–observation–sequencing datasets. In addition, applying these newer approaches to critical processes such as CO_2_ sequestration and fluxes in complex environments ranging from rainforest to alpine ecosystems is becoming not just an interesting research direction, but crucial to our own survival under global change. As biodiversity declines in response to human-driven change, what organisms, symbioses, and interactions are we losing? How are these shifts altering global biodiversity and ecosystem processes? The LCAs–mycorrhizal fungus–mycorrhizal plant model might be one such model system for further study.

## Data availability statement

The datasets presented in this study can be found in online repositories. The names of the repository/repositories and accession number(s) can be found below: https://www.ncbi.nlm.nih.gov/, PRJNA749330.

## Ethics statement

Ethical review and approval was not required for the study on animals in accordance with the local legislation and institutional requirements.

## Author contributions

The idea for this research came collectively from MA, PR, TH, EA, and HS. The first draft of the manuscript was written by MA, and reviewed and edited by PR, TH, HS, and EA. EA undertook the soil sampling, and HS undertook the sequencing. HS and EA completed the sequence analysis. All authors contributed to the article and approved the submitted version.
